# K^+^ and Rb^+^ Affinities of the Na,K-ATPase α_1_ and α_2_ Isozymes: An Application of ICP-MS for Quantification of Na^+^ Pump Kinetics in Myofibers

**DOI:** 10.3390/ijms19092725

**Published:** 2018-09-12

**Authors:** Hesamedin Hakimjavadi, Cory A. Stiner, Tatiana L. Radzyukevich, Jerry B. Lingrel, Natalie Norman, Julio A. Landero Figueroa, Judith A. Heiny

**Affiliations:** 1 Department of Pharmacology and Systems Physiology, University of Cincinnati, Cincinnati, OH 45229, USA; hakimjhn@mail.uc.edu (H.H.); stinerca@mail.uc.edu (C.A.S.); radzyutl@ucmail.uc.edu (T.L.R.); normanne@mail.uc.edu (N.N.); landerjo@ucmail.uc.edu (J.A.L.F.); 2 Department of Chemistry, University of Cincinnati, Cincinnati, OH 45221, USA; 3 Agilent Technologies Metallomics Center of the Americas, University of Cincinnati, Cincinnati, OH 45221, USA; 4 Department of Molecular Genetics, Biochemistry and Microbiology, University of Cincinnati, Cincinnati, OH 45229, USA; lingrejb@ucmail.uc.edu

**Keywords:** Na,K-ATPase, isozymes, skeletal muscle, potassium, rubidium, myofiber, affinity, ICP-MS

## Abstract

The potassium affinities of Na,K-ATPase isozymes are important determinants of their physiological roles in skeletal muscle. This study measured the apparent K^+^ and Rb^+^ affinities of the Na,K-ATPase α_1_ and α_2_ isozymes in intact, dissociated myofibers obtained from WT and genetically altered mice (α_1_^S/S^α_2_^R/R^ and skα_2_^−/−^). It also validates a new method to quantify cations in intact, dissociated myofibers, using inductively coupled plasma mass spectrometry (ICP-MS). Our findings were that: (1) The extracellular substrate sites of Na,K-ATPase bind Rb^+^ and K^+^ with comparable apparent affinities; however; turnover rate is reduced when Rb^+^ is the transported ion; (2) The rate of Rb^+^ uptake by the Na,K-ATPase is not constant but declines with a half-time of approximately 1.5 min; (3) The apparent K^+^ affinity of the α_2_ isozymes for K^+^ is significantly lower than α_1_. When measured in intact fibers of WT and α_1_^S/S^α_2_^R/R^ mice in the presence of 10 µM ouabain; the *K*_1/2*,K*_ of α_1_ and α_2_ isozymes are 1.3 and 4 mM, respectively. Collectively, these results validate the single fiber model for studies of Na,K-ATPase transport and kinetic constants, and they imply the existence of mechanisms that dynamically limit pump activity during periods of active transport.

## 1. Introduction

The Na,K-ATPase is an essential transport protein localized in the plasma membrane of all animal cells. The Na,K-ATPase catalyzes the efflux of three Na^+^ and influx of two K^+^ ions per molecule of ATP hydrolyzed, to maintain the steep transmembrane concentration gradients for Na^+^ and K^+^. The functional transporter consists of a primary catalytic α subunit, a β subunit and in most cells a regulatory FXYD subunit. Multiple isoforms of each subunit exist (α_1–4_, β_1–3_, and FXYD_1–7_) [1,2,3]; and provide a range of heteromers (isozymes) to serve the needs of different tissues and cells.

Mouse skeletal muscles express α_1_, α_2_, β_1_, β_2_, and FXYD1 (aka Phospholemman) subunits. Isozymes of α_2_ comprise up to 90% of total pump content in the mouse Extensor Digitorum Longus (EDL) [4]. This distribution in mammalian muscle differs from most other cell types which express α_1_ as the major, or sole, alpha subunit.

The α_2_ isoform serves a unique role in skeletal muscle [5]. It operates significantly below its maximum transport capacity in quiescent, non-contracting muscle but is rapidly stimulated upon the start of muscle use. Stimulation of α_2_ activity helps maintain excitation and contraction and opposes muscle fatigue [5]. In quiescent muscles, basal transport by the Na,K-ATPase is less than 5% of the maximum total Na,K-ATPase capacity [6]. Basal Na/K transport is mediated largely by the minor α_1_ isoform and is sufficient to maintain resting ion gradients and the resting membrane potential [7].

The ability of α_2_ to adjust its transport activity dynamically is conferred in part by the affinity of its extracellular facing substrate sites for K^+^ [8]. Muscle contraction is initiated by repetitive action potential activity, which transiently increases the extracellular K^+^ concentration ([K^+^]_o_). The largest excitation-related increase in [K^+^]_o_ occurs in the transverse tubules (t-tubules), where [K^+^]_o_ can reach tens of mM [9]. α_2_ isozymes have a lower apparent affinity for extracellular K^+^ (*K*_1/2*,K*_ = 4–5 mM) than α_1_ (*K*_1/2*,K*_ = 1–2 mM), and are present at high density in the t-tubules where they can sense and respond to increases in [K^+^]_o_ above resting concentrations. The lower K^+^ affinity of α_2_ keeps it operating below its maximum activity at resting [K^+^]_o_ (4 mM), conserving ATP, but allows it to be rapidly stimulated when [K^+^]_o_ rises. Because substrate stimulation is extremely rapid and not rate-limiting in the transport cycle [10,11], the lower K^+^ affinity of α_2_ provides a rapid mechanism for stimulating α_2_ activity to meet the increased demands of working muscle for [K^+^]_o_ uptake and [Na^+^]_i_ clearance.

Measurement of apparent substrate affinities in intact, living cells is always approximate and depends on the experimental model and conditions. In a previous study [8], we determined the K^+^ affinity of the α_2_ subunit isoform in muscle using two independent assays: the K^+^-dependence of pump currents measured in single, voltage-clamped mouse flexor digitorum brevis (FDB) fibers, and the K^+^-dependence of ATPase turnover measured using a sarcolemmal-enriched membrane preparation from mouse EDL. In both models, total Na,K-ATPase transport was activated by extracellular K^+^ and [K^+^]_o_ was varied to measure the K^+^ dependence of pump current or ATP turnover. The voltage clamp allowed us to measure the *K*_1/2*,K*_ of α_2_ in single fibers under passive conditions in which all other transport pathways were inhibited using ion replacement or blockers. Fixing the membrane potential prevented depolarization as [K^+^]_o_ was elevated, thereby avoiding both voltage-dependent pump stimulation and membrane potential changes associated with electrogenic pump transport. Those measurements used physiological resting concentrations of extracellular [Na^+^]_o_ and intracellular [K^+^]_i_ and a membrane potential near the physiological resting value (−90 mV). However, the intracellular ionic milieu was perturbed from resting conditions by the intracellular electrode solution. Intracellular [Na^+^]_i_ was raised to 80 mM, significantly above resting and contraction-related intracellular Na^+^ concentrations, to saturate the intracellular facing substrate sites, and intracellular Ca^2+^ was buffered with mM EGTA to prevent contraction. Na^+^ and K^+^ compete for shared intracellular- and extracellular-facing substrate sites on Na,K-ATPase and their concentrations interact to influence binding. The extent to which the K^+^ affinity of α_2_ may be influenced by intracellular [Na^+^]_i_ and/or excitation-related increases in [Ca^2+^]_i_ is not known.

An additional uncertainty in previous measurements of K^+^ affinities is that the contributions of α_1_ and α_2_ were identified pharmacologically using ouabain. In rodents, only the α_2_ isoform is inhibited by µM concentrations of ouabain. Pharmacological separation is reasonably good at resting [K^+^]_o_. However, the uncertainty increases as [K^+^]_o_ is raised because K^+^ has multiple actions on the Na,K-ATPase. K^+^ both activates transport and is itself transported, thereby dynamically changing the intracellular K^+^ concentration, and K^+^ decreases ouabain affinity in a concentration-dependent manner [12]. For these reasons and the low abundance of α_1_, the *K*_1/2*,K*_ of α_1_ could not be determined from measurements of pump current. Additional considerations apply to the ATPase turnover assay. With purified membranes, the sidedness of Na^+^ and K^+^ concentrations is lost and the membrane potential is depolarized to 0 mV.

The goals of this study were included: (1) Determining the apparent K^+^ affinities of the α_1_ and α_2_ isozymes in intact, non-voltage-clamped skeletal muscle fibers with an unperturbed intracellular environment, under ionic conditions as close to physiological as achievable, using genetic models to better isolate the contributions of α_1_ and α_2_ isoforms. We measured Rb^+^ uptake in samples of single EDL fibers of WT and genetically modified mice under conditions in which each has only a single active alpha isoform. Rb^+^ uptake was measured by Inductively Coupled Plasma Mass Spectroscopy (ICP-MS) as described [13]. (2) Comparing the affinities of Na,K-ATPase for Rb^+^ and K^+^, and evaluate the possibility of using Rb^+^ at mM concentrations as a K^+^ congener for studies of pump transport. (3) Improving the precision and time resolution of Rb^+^ uptake measurements, to obtain the time course and instantaneous transport rates of Na,K-ATPase. This was achieved by extending the Rb^+^ uptake assay to a multi-well format, to measure Rb^+^ uptake simultaneously in multiple samples of 2–5 single muscle fibers.

Our results show that the *K*_1/2_ of α_2_ isozymes for K^+^ in intact EDL fibers is 3.95 mM. This value is lower than previously determined from α_2_ pump currents using pharmacological separation alone. It is also significantly higher than the *K*_1/2*,K*_ of α_1_, consistent with previous reports. The affinity of Na,K-ATPase for Rb^+^ is identical to K^+^; however, Na,K-ATPase turnover is slowed when Rb^+^ is the transported cation. We further show that, at constant extracellular [K^+^]_o_, pump transport in intact fibers is not constant as commonly assumed, but decays with a time constant of 1–2 min This finding implies the existence of mechanisms that dynamically limit pump activity during periods of active transport.

## 2. Results

### 2.1. Detection Limits, Sample Size, and Selection of Elemental Mass Tag

Extracellular K^+^ is the natural substrate for cation uptake by the Na,K-ATPase. Rb^+^ (^86^Rb α β and γ emitter radioactive isotope or naturally abundant ^85^Rb, ^87^Rb) is commonly used as a K^+^ congener for measuring cation uptake in intact cells. Unlike K^+^, the endogenous intracellular Rb^+^ content of skeletal muscle fibers is extremely low, so that small increases in [Rb] produced by active Na,K-ATPase uptake can be detected above background.

**Figure 1:**
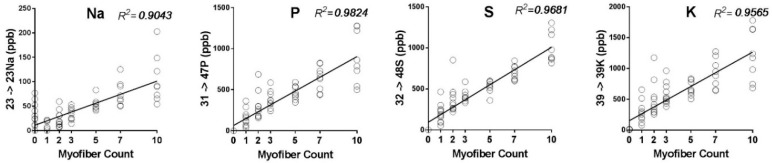

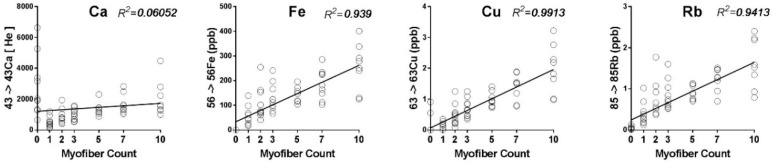
Basal concentrations (ppb) of 7 elements in samples of EDL fibers equilibrated in a standard (Rb-free) Tyrode’s solution. All measurements were performed simultaneously in a multi-well plate. Each symbol represents one sample from one well. Each sample contained 1–10 fibers. 3–6 samples were processed for each element and fiber count. Solid lines: linear regression fit to each data set.

Figure 1 evaluates the detection limits for measuring Rb^+^ in small samples of 1–20 dissociated fibers, and evaluates the use of Na, P, S, K, Fe, and Cu as potential elemental mass tags. The concentration of each element increases linearly with the number of fibers in the sample, as expected, with P, S, Ca, and K^+^ having the lowest *R*^2^ values. The variance also increases with fiber number, reflecting greater amounts of retained water in larger samples and differences in size of individual fibers. The minimum sample size for detecting these elements with a relative standard deviation less than 50% is two fibers. Samples of 2–5 fibers contain ≤1 ppb endogenous Rb^+^, allowing detection of exogenous Rb^+^ at ppb concentrations with a good signal-to-background ratio.

The linear relationships between element concentration and fiber number validates these elements as mass tags for normalization. S and P are useful for studies of Na,K-ATPase transport because they are abundant in skeletal muscle and are not transported by the Na,K-ATPase or by a secondary transporter which uses the concentration gradients generated by Na,K-ATPase activity as a driving force. For this study, we normalized Rb^+^ concentrations to the P content of each sample.

### 2.2. Optimization of the Wash Procedure

Measurement of Na,K-ATPase activity in small samples of muscle fibers requires stringent attention to removing all contaminating sources of Rb^+^ and the selected mass tag. To achieve this, we followed protocols developed previously for micro-samples [14] and further optimized the wash procedure. After incubation in a Rb^+^-containing Uptake Solution, the samples are washed several times in a Rb^+^-, Na^+^-, and K^+^-free wash solution at 0 °C to remove extracellular Rb^+^ and prevent further transport by the Na,K-ATPase. A relatively long wash procedure (four 15 min washes) was used for measurements in whole EDL muscles [13], to allow for diffusion through the complex interstitial spaces. Dissociated fibers do not have this diffusion limit.

**Figure 2:**
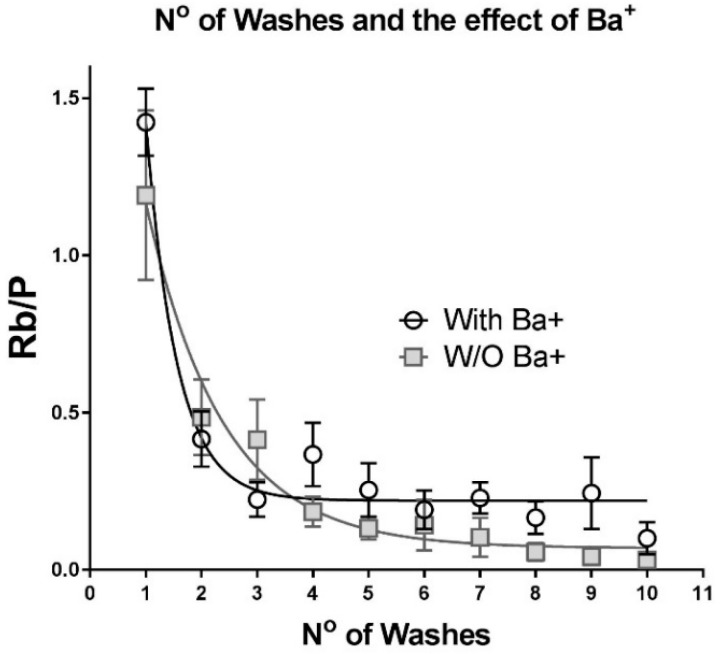
Residual Rb/P content in samples of dissociated fibers after 1–10 washes in a Rb^+^, Na^+^, and K^+^ free wash solution at 0 C. Samples of 2–5 fibers were incubated for 4 min in Rb-containing uptake Buffer, then washed from 1–10 times for 1 min each in standard Wash Solution (■), or Wash Solution containing 3 mM BaCl_2_ (○). Error bars show the SEM for *n* = 5–8 samples at each wash.

For this study, five washes of 1 min duration were sufficient to reduce extracellular Rb^+^ to insignificant levels (Figure 2). However, two sample populations were observed. In one group, residual Rb^+^ declined rapidly to a plateau level after four or five washes; while in another group, Rb^+^ continued to decline at a slower rate even after five washes. Addition of 3 mM BaCl_2_ to the wash solution preserved the initial decline and significantly reduced the slow component, suggesting that it reports outward Rb^+^ leak through a nonspecific K/Rb transport pathway that is not blocked in the standard wash solution. Thereafter, we standardized the wash protocol to five washes of 1 min with each using wash solution containing 3 mM BaCl_2_.

### 2.3. Na,K-ATPase Mediates the Majority of Active Rb^+^ Uptake in This Assay

**Figure 3:**
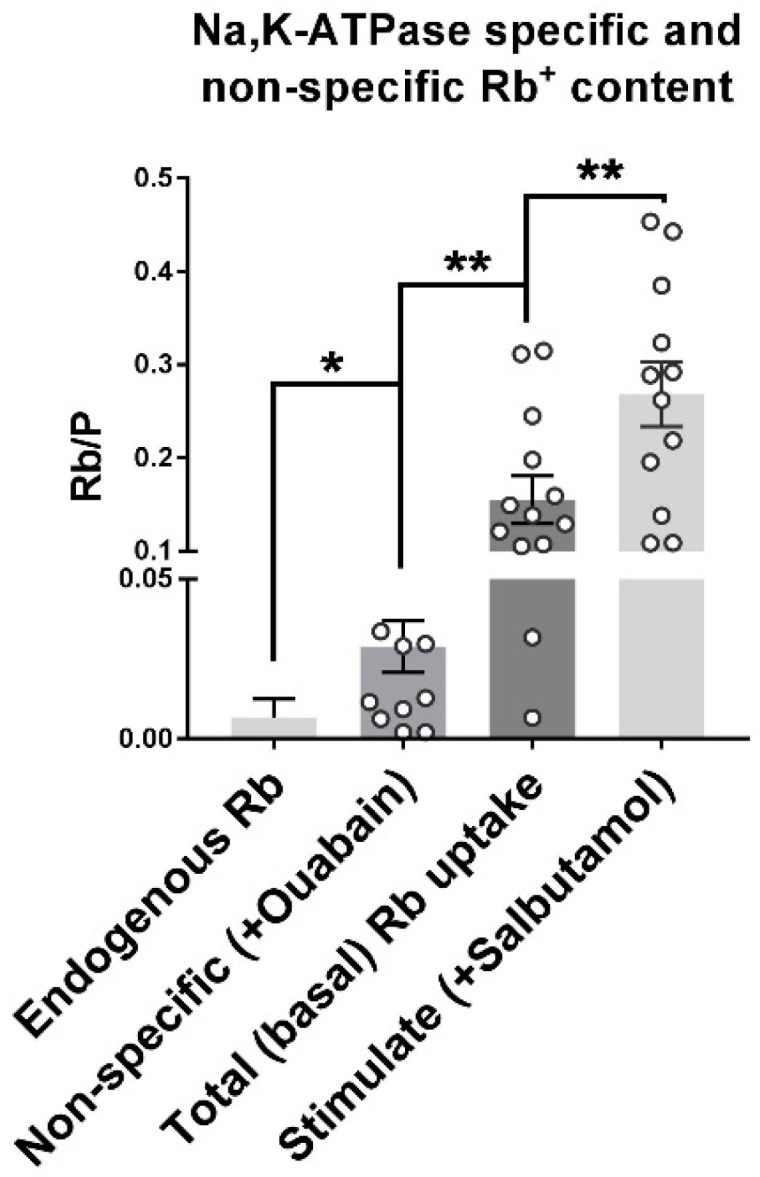
Rb^+^ uptake by the Na,K-ATPase measured by ICP-MS in mouse EDL fibers. Samples of 2–5 fibers were incubated for 4 min in each of four solutions: Rb-free Tyrode’s (endogenous Rb^+^ content); uptake solution containing 500 µM RbCl, 5.5 mM K^+^ and 1 mM ouabain (non-specific Rb^+^ uptake); uptake solution without ouabain (total basal Rb^+^ uptake in quiescent fibers); or uptake solution containing an agonist (10 µM salbutamol) of Na,K-ATPase transport (stimulated Rb^+^ uptake). Rb^+^ concentrations were normalized to the P concentration of each sample, Rb/P. Each symbol represents a measurement from one sample. Each group included 12–13 samples (total: 24–65 fibers per group). All measurements were performed simultaneously in a multi-well platform. * and ** indicate significant difference of mean values at *p* < 0.05 and *p* < 0.01, respectively.

Specific Rb^+^ uptake by the Na,K-ATPase is determined as shown in Figure 3. The average absolute endogenous Rb^+^ concentration of samples of 2–5 fibers in standard Tyrode’s was 1.58 ± 1.28 ppb. The minimum uptake period for resolving exogenous Rb^+^ above background was 4 min Specific uptake by the Na,K-ATPase is identified using an inhibitor (ouabain) or an agonist (salbutamol) of Na,K-ATPase transport [15]. The P content of each sample is used as a mass tag for normalization. The use of elemental mass tags significantly reduces the within-group variance and increases the power of the measurement compared to normalization by wet weight [13] or number of fibers. Moreover, accurate measurement of sample weight is not possible with microsamples. Normalization to P content reduced the relative standard deviation (RSD) of passive, total and stimulated Rb^+^ uptake from 114, 63 and 50% to 97, 60 and 44% respectively (Table 1).

Non-specific Rb^+^ taken up by 2–5 fibers incubated for 4 min in Uptake Solution containing 500 µM tracer Rb^+^, 5.5 mM K^+^, and 1 mM ouabain is 0.03 ± 0.02 Rb/P. In quiescent fibers, total Rb^+^ uptake is 0.16 ± 0.09 Rb/P under basal conditions, and 0.28 ± 0.12 Rb/P in the presence of salbutamol. Specific Na,K-ATPase uptake represents approximately 80% of total Rb^+^ uptake in these conditions. Na^+^ pump-specific Rb^+^ uptake can be measured reliably with a S/N > 5.5.

### 2.4. Extracellular Substrate Affinity of Na,K-ATPase for Rb^+^ is Identical to K^+^, but Maximum Turnover Rate is Slower when Rb^+^ is the Transported Ion

**Figure 4:**
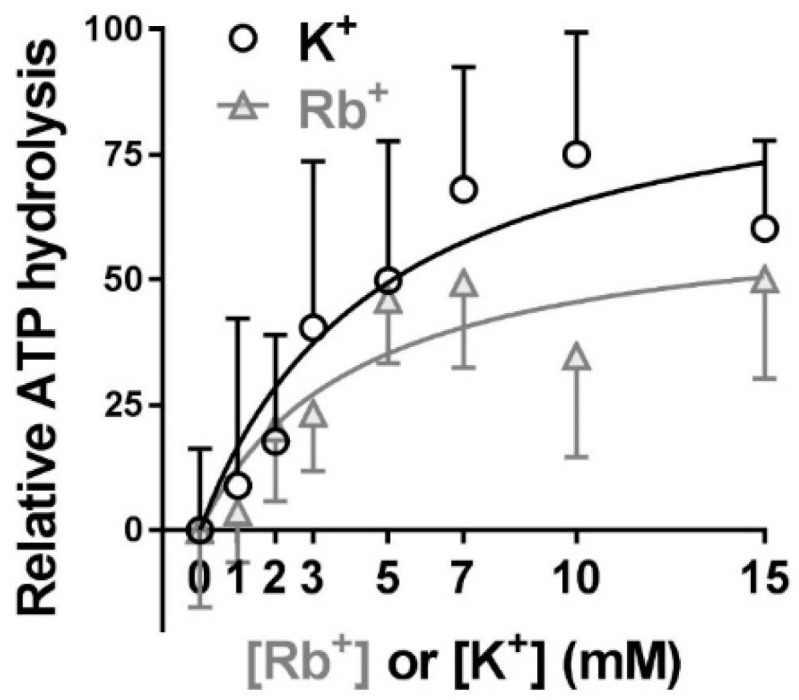
Substrate affinities of the Na,K-ATPase for K^+^ and Rb^+^. Na,K-ATPase activity was measured using an ATPase turnover assay and a sarcolemmal-enriched membrane preparation obtained from WT mouse EDL. K^+^ or Rb^+^ concentrations were varied by equimolar replacement with choline chloride at constant osmolarity. [Na^+^]_i_ was fixed at 80 mM to saturate substrate stimulation by Na^+^, and [K^+^] or [Rb^+^] was varied from 1–15 mM. Each symbol reports the mean and SEM of triplicate measurements on 3–4 samples at the indicated concentration (total of 32 and 31 samples for Rb^+^ and K^+^, respectively). Solid lines show a best fit of the data to the Michaelis–Menten equation (Table 2).

Figure 4 evaluates whether Rb^+^ can be used at mM instead of tracer concentrations in the uptake solution, to increase the signal-to-background ratio. K^+^ is the natural substrate for Na,K-ATPase. When extracellular Rb^+^ is limited to tracer concentrations, the measured substrate affinity reflects K^+^ affinity. Using RbCl at higher concentrations to obtain the apparent K^+^ affinity is valid if the affinities of Na,K-ATPase for Rb^+^ and K^+^ are similar. However, the affinity of Na,K-ATPase for Rb^+^ has not been measured previously in muscle.

To address this question, we compared the affinities of Na,K-ATPase for Rb^+^ or K^+^ using an ATPase turnover assay and a sarcolemmal-enriched membrane preparation (Figure 4). This approach allowed us to determine the intrinsic K^+^ affinity and *V_max_* of each cation in a minimal, well-validated model. In this assay, each cation has access to the substrate sites without diffusion delays and the concentrations of each substrate remain constant even at very high transport rates. This condition is not achievable in intact muscles.

The Rb^+^ and K^+^ dependence of ATPase activity both show saturation, as expected for an active transport mechanism with substrate binding. The apparent K^+^ affinity of Na,K-ATPase is not significantly different from the apparent affinity for Rb^+^ binding. However, the maximum turnover rate is reduced 1.5-fold when Rb^+^ replaces K^+^ as the transported ion. This result indicates that Rb^+^ and K^+^ bind to the extracellular substrate sites of Na,K-ATPase with comparable affinities; however, the maximum turnover rate is slowed when Rb^+^ is the transported ion. Therefore, Rb^+^ at mM concentrations can be used as a K^+^ congener to obtain the extracellular substrate affinity, but not *V_max_*, of Na,K-ATPase.

### 2.5. K Affinity of Na,K-ATPase Measured in Dissociated Fibers

**Figure 5:**
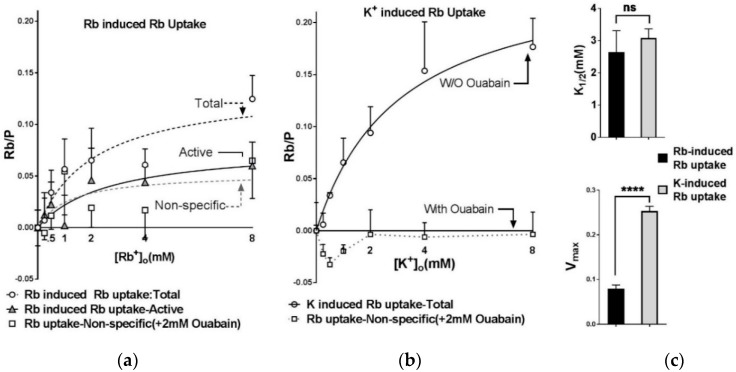
Apparent K^+^ affinity of Na,K-ATPase determined from measurements of Rb^+^ uptake in dissociated, intact EDL fibers of WT mice. (**a**) Rb-activated Rb^+^ uptake. Samples of 2–5 fibers were incubated for 4 min in uptake solution containing 0 to 8 mM RbCl, in the absence of extracellular K^+^. The extracellular Rb^+^ concentration was varied by equimolar replacement with choline chloride at constant osmolarity. Prior to the uptake period, samples were pre-incubated for 5 min in a Rb- and K-free Tyrode’s solution without (○) or with 2 mM ouabain (□). Uptake was initiated by changing to the Rb-containing uptake solutions. The subtracted data at each [Rb^+^]_o_ (▲) represents the ouabain-sensitive, Na,K-ATPase component of Rb^+^ uptake. Each symbol represents the mean and SEM of 40 and 37 samples for active and non-specific Rb-induced uptake, respectively. Solid and dashed lines represent best fit curve to the Michaelis–Menten equation. (**b**) K-activated Rb^+^ uptake. Same protocol for uptake solution containing 0–8 mM K^+^ and 500 µM Rb^+^ (tracer). Each symbol represents the mean and SEM of 38 and 39 samples for active and non-specific K-induced Rb^+^ uptake, respectively). (**c**) The extracellular substrate affinities for Rb^+^ and K^+^ are not significantly different when measured in dissociated fibers (*p* = 0.783); however, *V_max_* is significantly less when Rb^+^ is the major transported cation (*p* < 0.0001).

To further investigate this question, we compared the affinity of Na,K-ATPase for K^+^ and Rb^+^ in dissociated fibers (Figure 5). Using extracellular Rb^+^ at mM concentrations revealed a large component of Rb^+^ uptake that increases with the Rb^+^ gradient (Figure 5a). This component remains in the presence of 2 mM ouabain, suggesting the existence of a passive, ouabain-insensitive Rb^+^ leak pathway that operates when the Rb/K concentration gradients are preserved. A gradient-driven passive Rb^+^ uptake would not be seen in membrane models which lack substrate concentration gradients. The ouabain-inhibited component of active Rb-induced Rb^+^ uptake, reflecting NKA-specific active Rb^+^ transport, fit to the Michaelis–Menten equation for *K*_1/2,Rb_ = 2.65 ± 4.18 mM.

The ouabain-insensitive component of Rb^+^ leak is negligible when Rb^+^ is used at tracer concentrations (500 µM) (Figure 5b). In this condition, The *K*_1/2*,K*_ for Na,K-ATPase uptake obtained from measurements of K-induced (tracer Rb^+^) uptake is 3.08 ± 1.78 mM, and is not significantly different from the affinity obtained from measurements of Rb-induced Rb^+^ uptake (*p* = 0.782).

On the other hand, *V_max_* is 2.6-fold greater when measured using K^+^-induced compared to Rb-induced Rb^+^ uptake (Figure 5c). This difference is in the same direction, but greater, than measured using the homogenate system. Collectively the results shown in Figure 4 and Figure 5 suggest that Rb^+^ and K^+^ bind to the extracellular substrate sites of Na,K-ATPase with comparable affinities. However, turnover is slowed when Rb^+^ is the transported ion.

### 2.6. Instantaneous Rate of Rb^+^ Uptake by the Na,K-ATPase Is Not Constant

Measurements of Rb^+^ uptake by intact cells are commonly used to obtain transport rates of the Na,K-ATPase. Transport rates are computed as the amount of Rb^+^/K^+^ taken up divided by the uptake period, and reflect the average transport rate over the uptake period. This calculation assumes that pump transport is constant during the measurement. However, real-time measurements of pump current suggest that this assumption may not be valid [8].

**Figure 6:**
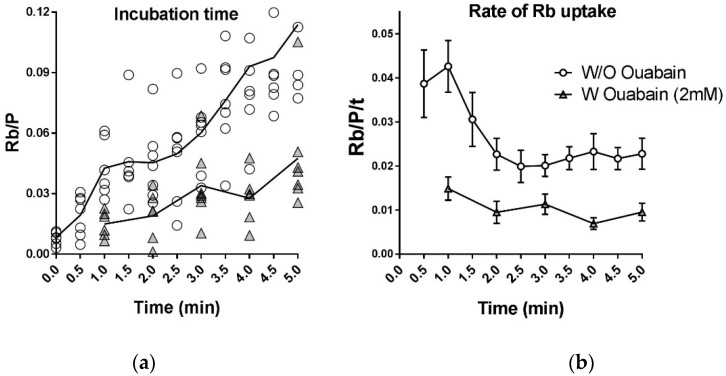
Rate of Rb^+^ uptake by Na,K-ATPase in intact fibers measured using 30 s intervals. (**a**) The amount of Rb^+^ taken up by samples of 2–5 fibers for incubation times of 0.5 to 5 min Uptake was initiated at time 0 by perfusing the samples with uptake solution containing 5.5 mM K^+^ and 500 µM RbCl (35 C). Uptake was stopped at the indicated times by changing to wash solution and applying the wash protocol described in Figure 2. Symbols: (○) total Rb^+^ content at each time point in standard Rb^+^ uptake solution. (▲) passive (ouabain-insensitive) Rb^+^ uptake in the presence of 2 mM ouabain. *Y*-axis: Rb/P without subtraction of endogenous Rb^+^ content; (**b**) average uptake rates, *ΔRb*/*p*/*Δt*, computed for each 30 s interval and plotted at end of each interval. The Rb^+^ content of each sample was normalized to P content, Rb/P.

To test this assumption, we measured Rb^+^ uptake by the Na,K-ATPase in dissociated fibers using shorter uptake intervals (Figure 6). The amount of Rb^+^ in the samples increases steadily as the uptake period is increased, as expected (Figure 6a). Notably, the Rb^+^ uptake rate, *ΔRb*/*Δt*, is not constant (Figure 6b). It is greatest immediately after uptake is initiated and then decreases to a steady level over the next 2–3 min Non-specific Rb^+^ uptake rate (▲symbols) is relatively constant over the same period.

Practically, this result suggests that an uptake period of 3–5 min is optimal for measurements comparing Rb^+^ uptake by dissociated myofibers in different conditions. In this period, the rate of Rb^+^ uptake is relatively steady while the ratio of total-to-nonspecific Rb^+^ uptake is greatest. Thereafter, we used a standard uptake period of 4 min

### 2.7. K Affinity of α_1_ and α_2_ Isoforms of Na,K-ATPase in Dissociated Fibers

**Figure 7:**
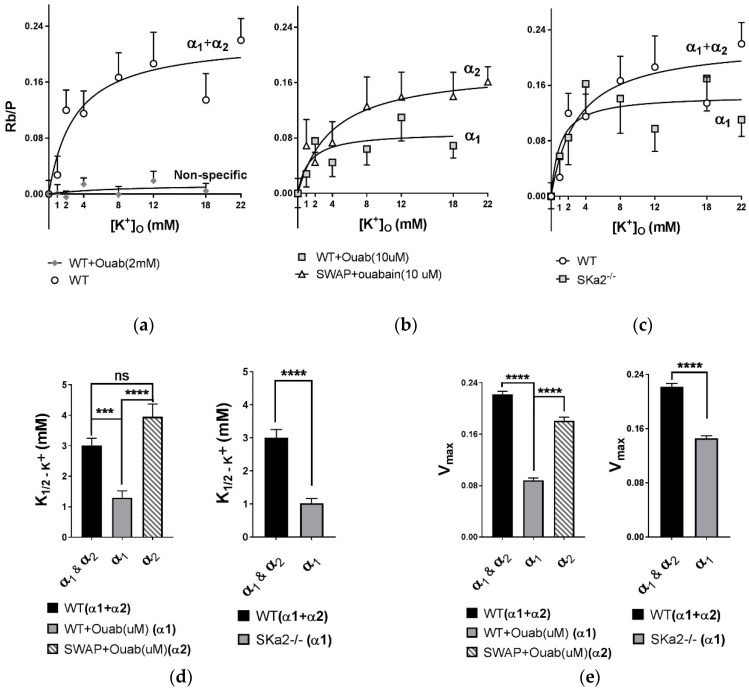
Apparent K^+^ affinities of α_1_ and α_2_ isozymes of Na,K-ATPase obtained from measurements of tracer Rb^+^ uptake in EDL fibers of WT, ‘SWAP’ and skα_2_^−/−^ mice. Samples of 2–5 fibers were incubated for four min in uptake buffer containing 0 to 20 mM [K^+^]_o_ and 500 µM RbCl, and washed as described in Figure 2. The samples were pre-incubated for 5 min in Rb^+^ and K^+^ free buffer containing 0, 10 µM, or 2 mM ouabain. Total Rb^+^ content is normalized to the P content of each sample. (**a**) K^+^ dependence of Rb^+^ uptake in WT fibers in the absence (both α_1_ + α_2_ remain active) and presence of 2 mM ouabain (no active Na,K-ATPase isoform). *N* = 46 and 41 samples, respectively; (**b**) K^+^ dependence of Rb^+^ uptake in WT or ‘SWAP’ fibers in the presence of 10 µM ouabain (α_1_ is active in WT, α_2_ is active in ‘SWAP’; *n* = 39 and 46 samples, respectively); (**c**) K^+^ dependence of Rb^+^ uptake in WT and skα_2_^−/−^ fibers ±2 mM ouabain (α_1_ only, at 2–3-fold increased abundance). *N* = 46 and 46 samples, respectively. Note: the curve for WT in 7c is the same as 7a and is superimposed on skα_2_^−/−^ data for comparison; (**d**,**e**) *K*_1/2*,K*_ and *V_max_* for each genotype, obtained from a fit of the data sets in a–c to the Michaelis–Menten equation.

The measurements reported in Figure 3, Figure 4, Figure 5 and Figure 6 represent K^+^ or Rb^+^ uptake by the total muscle pool of Na,K-ATPase, which includes both α_1_ and α_2_ isozymes. The nonspecific component of uptake was obtained using mM ouabain to inhibit both isoforms. To measure the separate K^+^ affinities of the α isoforms in dissociated fibers, we measured Rb^+^ uptake in EDL fibers from WT and α_1_^S/S^α_2_^R/R^ (aka ‘SWAP’) mice, each in the presence of µM ouabain (Figure 7). In this condition, α_1_ is the sole active isoform in WT fibers and α_2_ is the sole active isoform in ‘SWAP’ fibers. This experimental model preserves the natural abundance of each α isoform as well as the physiological gradients for Na^+^ and K^+^. Intracellular Na^+^ is not perturbed from resting levels, and the same low concentration of ouabain is used to block either isoform. Under these conditions, *K*_1/2*,K*_ of α_1_ is 1.30 ± 1.40 mM, and *K*_1/2*,K*_ of α_2_ is 3.95 ± 2.85 mM (*p* < 0.0001) (Figure 7d and Table 3). *V_max_* of α_2_ transport is twofold greater than α_1_ (0.18 vs. 0.09 Rb/P). These parameters reflect the K^+^ affinities and relative transport capacities of α_1_ and α_2_ isozymes under conditions of maximal extracellular substrate site occupancy (20 mM [K^+^]_o_) and resting [Na^+^]_i_ (10–11 mM).

Another approach to determine the affinity of α_2_ is to measure the K^+^ dependence of α_2_ in fibers of skα_2_^−/−^ mice (skeletal muscle-targeted α_2_ knock-out). α_1_ is the sole alpha isoform in skeletal muscles of these mice and its abundance is upregulated 2.5-fold [5], thereby facilitating its detection above background. Despite a large variance, *K*_1/2*,K*_ for α_1_ is 1.01 ± 0.95 mM in this model, consistent with the α_1_ affinity measured in WT fibers in the presence of µM ouabain (0.09 ± 0.02 mM).

## 3. Discussion

Knowledge of the substrate affinities of Na,K-ATPase isozymes is important for understanding their physiological roles and regulation. This study measured the kinetics of Rb/K transport by Na,K-ATPase in skeletal muscle and the apparent affinities of α_1_ and α_2_ isozymes for K^+^ and Rb^+^. Rb^+^ uptake was measured in microsamples of 2–5 intact, dissociated EDL fibers using a multi-well format that allowed parallel measurements on up to 96 samples. The Rb^+^ content of each sample was quantified by ICP-MS. The contributions of α_1_ and α_2_ isozymes to total Rb^+^ uptake were separated using WT mice and genetically altered mice under conditions in which only a single α isoform is active (WT or α_1_^S/S^α_2_^R/R^, aka ‘SWAP’ mice in the presence of 10 µM ouabain).

The main findings from this study are:

1. The apparent affinities of Na,K-ATPase α_1_ and α_2_ isoforms for K^+^, measured in intact muscle fibers with unperturbed intracellular environment, are 1.3 and 4 mM, respectively. These affinities are not significantly different from the K^+^ affinities of α_1_ and α_2_ isozymes measured in mouse skeletal muscles using other experimental models [8].
2. The extracellular substrate sites of Na,K-ATPase bind Rb^+^ and K^+^ with the same apparent affinity. However, pump turnover rate is reduced when Rb^+^ is the transported ion. Practically, this means that Rb^+^ can be used at mM concentrations as a congener for K^+^ for determining extracellular substrate affinity but should be limited to tracer concentrations to accurately measure *V_max_* of Na,K-ATPase.
3. The instantaneous rate of K/Rb transport is not constant but declines to a steady level with a half time of 1–2 min Therefore, Rb^+^ uptake measured using uptake periods of ≤30 s reports a near-peak uptake rate, and Rb^+^ uptake measured for uptake periods >5 min reports a near steady-state uptake rate.
4. Measurement of Rb^+^ uptake in intact, dissociated fibers using a multi-well format and quantification of Rb^+^ content by ICP-MS provides a useful, intermediate throughput assay with good time resolution and statistical power. The use of a WT mice and transgenic mice with altered ouabain affinities allowed us to measure the separate contributions of α_1_ and α_2_ isozymes using a single, low concentration of ouabain (10 µM). This strategy provided a better separation of isoform-specific contributions than was possible using WT fibers alone and a 1000-fold range of ouabain concentrations (µM and mM), in which the overlap in ouabain affinities of native α isoforms contributes some overlap to their measured K^+^ affinities.

Collectively, these results further validate the multi-well assay of Rb^+^ uptake in microsamples of dissociated muscle fibers.

### 3.1. Isoform-Specific Affinities for Extracellular K: Physiological Implications

The finding that α_2_ isozymes of Na,K-ATPase bind extracellular K^+^ with a significantly lower apparent affinity than α_1_ isozymes is consistent with a previous study of their K^+^ affinities measured in voltage-clamped mouse FDB fibers, and in a muscle sarcolemmal membrane preparation [8]. Together, these studies strengthen the conclusion that the lower affinity of α_2_ for extracellular K^+^ renders α_2_ isozymes less active in quiescent, non-contracting muscles, and more active during periods of muscle use. The lower K^+^ affinity and localization of α_2_ in the transverse tubules position it to respond to the excitation-related increases in extracellular K^+^ that occur during periods of muscle use.

The K^+^ affinity of α_2_ reported in this study reflects the combined affinity of the two possible α_2_ isozymes, α_2_β_1_ and α_2_β_2_. Studies on recombinant Na,K-ATPase pumps have shown that the β subunit isoform influences the K^+^ affinity of the functional enzyme [16]. In particular, the β_2_ subunit lowers the K^+^ affinity of the isozyme, with α_2_-β_2_ isozymes having the lowest K^+^ affinity (3.5 mM) of the possible α-β dimers [17]. The β subunit partner of α_2_ in mouse muscle t-tubules is not known. Mouse EDL/fast muscles express both β_1_ and β_2_ [18]. In rat muscles, β_2_ is the major expressed isoform in fast muscles and β_1_ is the predominant isoform in slow muscles [19]. The affinity of α_2_ isozymes measured in mouse EDL and FDB fast muscles (4–4.3 mM) is close to the lowest measured affinity of rat recombinant isozymes (α_2_-β_2_ [17]). These considerations suggest that the K^+^ affinity of α_2_ isozymes reported in this study reflects largely α_2_-β_2_ isozymes.

Table 4 compares the K^+^ affinities of α_1_ and α_2_ isozymes in mouse fast skeletal muscles, obtained using different experimental models. Overall, measurements of the extracellular substrate affinities are consistent over a wide range of experimental models and conditions. The affinity of α_2_ for K^+^ is expected to be slightly higher when measured in membranes than in intact fibers. In membrane preparations, the lower-than-physiological extracellular Na^+^ concentration (70 mM) and depolarized membrane potential (0 mV) both favor Na^+^ unbinding from the shared sites in the E2 conformation and thereby facilitate K^+^ binding. The K^+^ affinity of α_1_ shows a large variance in all studies and is attributed to the lower abundance and minor contribution of α_1_ to total pump activity in skeletal muscle. A value of 1–2 mM for α_1_ is reported in a wide range studies using different tissues and species [8,17,19,20].

### 3.2. Use of Rb^+^ as a Congener for K^+^ in Studies of Na,K-ATPase

K^+^ is the natural substrate for Na,K-ATPase. At maximal extracellular substrate site occupancy, pump turnover is slowed when Rb^+^ replaces K^+^ as the transported cation. A slower transport rate for Rb^+^ was also reported in measurements using whole EDL muscles [13]. It is possible that conformational transitions in the transport cycle subsequent to K^+^ binding are altered when the larger Rb^+^ ion is bound in place of K.

### 3.3. Na,K-ATPase Transport Rates Decline in Intact Fibers under Dynamically Changing Substrate Conditions

Transport rates obtained from measurements of Rb^+^ uptake, computed as the amount of Rb^+^ taken up divided by the uptake period, reflect the average transport rate during the uptake period. The calculation assumes that transport rate is constant during the measurement. This assumption is valid if both extracellular K/Rb and intracellular Na^+^ concentrations remain constant, and if intrinsic mechanisms that modulate substrate affinity or pump activity are absent.

The requirement for equilibrium substrate concentration at the extracellular binding sites is difficult to achieve in whole muscles. Incubation times of 5–15 min are commonly used to ensure a constant extracellular [K]_o_ in the interstitium of whole muscles. Single myofibers lack this diffusion limit but retain a delay of ≤3 s for diffusion of small ions into the t-tubules [21,22]. This small delay is not expected to contribute significantly to transport rates measured using 30 s uptake periods.

The unexpected finding in these measurements is that, even when extracellular substrate concentration is reasonably constant, the transport rate is not. Rather, transport activated by constant 4 mM [K^+^]_o_ declines to a plateau with a half time of 1–2 min (Figure 6). The physiological basis of the decline is not known. A decay in pump current within the first min of pump activation was also seen in measurements of pump currents in mouse FDB fibers, and was attributed to an inward, time-dependent leak that may partly mask outward pump current [8]. However, non-specific uptake in the current study is constant (Figure 6b), consistent with a time-independent, passive transport process.

A decay in pump current is also seen in cardiac myocytes [23,24]. The decline in myocytes occurs in two phases—a fast component that is complete in <1 s and a slower component that decays in tens of seconds depending on the intracellular [Na^+^]_I_ [24]. The fast component is attributed to an intrinsic mechanism of pump inactivation and the slow component is attributed to decreased substrate activation by intracellular [Na^+^]_i_ as Na^+^ is actively extruded [24].

The decline in pump transport seen in this study, which occurs over minutes, may reflect, in part, de-activation of pump transport by depletion of intracellular Na^+^ during active Na^+^ extrusion. The intracellular Na^+^ concentration is not controlled in intact, dissociated fibers. Operating at maximum speed, the Na,K-ATPase cycles at 800/min and extrudes 3 Na^+^ ions from a small, closed volume of approximately 8 µL [25]. Consequently, [Na^+^]_i_ may change dynamically during active transport, with the greatest decrease expected at high transport rates. Depletion of [Na^+^]_i_ occurs in C2C12 myocytes during repetitive electrical stimulation [26]. A quantitative assessment of the extent to which intracellular substrate depletion occurs in skeletal muscle under these conditions will require measurements in which [Na^+^]_i_ is controlled or measured simultaneously with pump activity.

### 3.4. Intermediate throughput Assay of Na,K-ATPase Activity in Intact Muscle Cells

The workable range for measuring Rb^+^ uptake by ICP-MS in this study is in the low ppb range. This range was calculated based on the amount of Rb taken up by a sample of 2–5 fibers under resting conditions during a 4 min incubation in 500 µM RbCl (20–60 ppm), compared to the endogenous Rb concentration of the sample (~1 ppb). A ppb range was achieved by using an elemental mass tag for normalizing Rb^+^ uptake by samples having different numbers of fibers of varying sizes; and by optimizing the protocol to reduce extraneous sources of Rb^+^ and other metals, as described in this and a previous study. In addition to P, we validated S, Na, K, Fe, Cu, and Rb^+^ as useful elemental tags for muscle mass. Having a choice of mass tags provides options for studying a range of physiological processes. For this study, we used P because it is abundant in skeletal muscles and is not transported by the Na,K-ATPase, or by a secondary transporter which uses the concentration gradients generated by the Na,K-ATPase as a driving force. Although inorganic phosphate (Pi) is actively transported into myofibers through type III sodium-phosphate (Na-Pi) cotransporters, the influx of Pi through Na-Pi transport is negligible on the time scale of our measurements. Rat type II muscles take up Pi at a rate of approximately 25 nM/g/min [27], whereas the Pi content of muscle fibers is in the mM range. The RSD of Rb^+^ uptake measured in 6 samples of 2–5 fibers from the same animal was 67%, compared to an RSD of 97% in measurements of Rb^+^ uptake in different animals. The RSD can be further reduced by increasing the number of fibers per sample and/or the number of replicates in each condition.

The method described in this study has significant advantages over conventional assays of Rb^+^ uptake in skeletal muscle. Conventional assays provide a single measurement from each sample, thereby requiring a large number of animals to probe multiple time points and conditions. The multi-well format allows parallel measurements on multiple samples from the same animal, thereby reducing animal-to-animal variability. The ability to process samples at the same time after surgical removal and fiber dissociation reduces differences in fiber condition. A range of experimental conditions can be studied simultaneously—e.g., control and ouabain-treated samples at each K^+^ concentration—while reducing variance in other factors such as temperature that influence pump transport. In this study, we harvested only superficial fibers and routinely obtained 20–50 viable fibers from two EDL muscles of one animal. A greater number of fibers can be obtained by prolonging the enzymatic dissociation step.

Importantly, the ability of ICP-MS to quantify multiple metals simultaneously will allow future studies of the relationship between Na,K-ATPase transport and the transport of other physiologically important ions. It is known that the Na^+^ concentration gradient generated by Na,K-ATPase activity provides the driving force for many secondary transporters, such as the Na-Ca Exchanger. However, the coupling relationship between the Na,K-ATPase and secondary transporters is incompletely understood, largely because of the difficulty of studying their coupling using radioactive tracers.

A summary of key differences between the new method and other conventional measurements of Na,K-ATPase activity is given below.

#### 3.4.1. Method: ATPase Hydrolysis Assay

- **Measurement**: Inorganic phosphate released by ATP hydrolysis, detected by fluorimetry.
- **Sample**: Sarcolemmal-enriched membranes prepared from skeletal muscle muscles.
- **Advantages**: (a) High reproducibility; (b) substrate concentrations at both extracellular and intracellular facing substrate sites can be fixed; (c) enzyme activity is computed using a single equation with simple stoichiometry.
- **Disadvantages:** (a) Low yield, purified membranes may not reflect the in vivo distribution of enzyme subunits; (b) physiological sidedness of substrate concentrations is absent.

#### 3.4.2. Method: Na,K-ATPase Specific Current (I_pump_)

- **Measurement**: Outward pump current.
- **Sample**: Dissociated, intact FDB fibers.
- **Advantages**: (a) Continuous recording of pump current with millisecond time resolution. (b) Extracellular [K]o can be fixed within seconds. (c) Intracellular [Na^+^]_i_ can be reasonably fixed by the pipette [Na^+^]_i_, due to the high pipette-to-fiber volume ratio. (d) V_m_, which influences substrate binding and transport activity, can be fixed.
- **Disadvantages:** (a) Passive conditions with all other ion transport pathways inhibited are non-physiological; possible interactions between Na,K-ATPase and other transport pathways are lost. (b) Regulation of pump activity by intracellular Ca^2+^ is prevented by the high concentrations of Ca^2+^ buffers used to block fiber contraction.

#### 3.4.3. Method: Rb Uptake in whole Muscles Measured by ICP-MS

- **Measurement**: ^85^Rb/^87^Rb uptake.
- **Sample**: Isolated whole muscle.
- **Advantages**: (a) Rb uptake is a ‘gold standard’ assay to measure Na,K-ATPase activity in intact tissues and cells with unperturbed intracellular milieu. (b) Close to physiological conditions with intact transport pathways for other ions. Studies of other transport pathways which operate in parallel or in concert with Na,K-ATPase are possible with ICP-MS. (c) Physiological resting Na^+^ and K^+^ ion gradients and membrane potential are preserved. (d) Intracellular environment, including the [Na^+^]_i_ is unperturbed. (e) Uses non-radioactive, naturally abundant ^85^Rb or ^87^Rb. (f) Rb is easily detected by ICP-MS due to low interference from other ions. (g) High sensitivity (1 part per billion detection limit).
- **Disadvantages:** (a) Extracellular diffusion delays through the muscle interstitium limit control of extracellular concentrations and increase Rb^+^ leak during extended wash times. (b) Compared to dissociated fibers, higher endogenous ionic background and greater non-specific Rb^+^ uptake by other transport pathways. (c) Limited to a single data point per muscle, increases variance and number of animals. (d) Rb leak during washing procedure.

#### 3.4.4. Method: Rb Uptake by Dissociated Single Fibers Measured with ICP-MS in a Multi-Well Format

- **Measurement**: ^85^Rb/^87^Rb uptake.
  **Sample**: Dissociated, intact fibers.
- **Advantages**: (a) Essentially no diffusion delays, increased time resolution. (b) Unperturbed intracellular milieu, closer to in vivo conditions in which [Na^+^]_i_ changes dynamically during Na,K-ATPase transport. (c) Medium throughput. (d) Parallel measurements under multiple conditions using fibers from the same animal greatly increases the statistical power. Most useful for dose dependent and time dependent studies.
- **Disadvantages:** (a) Possible selection bias—only resilient myofibers tolerate the dissociation procedure. (b) Extracellular matrix is compromised by enzymatic dissociation, with unknown consequences on transport pathways. (c) Increases nonspecific membrane leak.

#### 3.4.5. Method: Quantification of Na^+^ and K^+^ Content by Flame Photometry

- **Measurement**: Total Na^+^ and K^+^ content.
  **Sample**: Isolated whole muscle.
- **Advantages**: (a) Direct measurement of native cations Na^+^ and K^+^ (vs. tracer cations e.g., Rb^+^ and Li^+^). (b) High sensitivity and specificity for Na+. (c) Simulations quantification of Na^+^ and K^+^ enables determination of net ionic fluxes and changes in intracellular ion content directly. (d) An established method with several available trouble-shooting guides.
- **Disadvantages:** (a) High background [K]_i_ makes it difficult to detect the relatively small K influx. (b) K^+^ leaks from cells faster than Na^+^ during consecutive washes, which may introduce noise in the K^+^ reading. (c) In milli molar K^+^ concentrations, the read out of the flame photometric becomes non-linear regarding K^+^ concentration in solution, which makes it more difficult to use the assay in the millimolar range.

## 4. Materials and Methods

### 4.1. Animals

Adult wild-type (WT) male mice (C57BL/6; The Jackson Laboratory) or genetically altered mice (α_1_^SS^α_2_^RR^ aka ‘SWAP’) mice [28], and skα_2_^−/−^ [5] mice of 2–4 months of age were used as a source of tissue. The α_2_ gene (Atp1α_2_) is knocked out in the skeletal muscles of skα_2_^−/−^ mice. In ‘SWAP’ mice the natural affinities of the α_1_ and α_2_ isoforms are reversed. The α_1_ isoform, which in rodents is normally resistant to ouabain binding, is made sensitive to ouabain binding, and the normally sensitive α_2_ isoform is made resistant. This model was created by changing two amino acids in each isoform without altering their expression levels or transport properties [28]. These models make it possible to identify the contributions of α_1_ and α_2_ isozymes to pump function using the same, low µM concentration of ouabain in all assays. Mice were anesthetized (2.5% Avertin, 17 mL/kg) during tissue extraction and euthanized after tissue removal. All procedures were performed in accordance with the Guide for the Care and Use of Laboratory Animals (National Research Council of the National Academies, USA) and were approved by the Institutional Animal Care and Use Committee of the University of Cincinnati (IACUC approval #05-07-08-01, 30 June 2016).

### 4.2. Single Fiber Dissociation

Single muscle cells (fibers) were obtained using a modification of a published protocol [8]. Two extensor digitorum longus (EDL) muscles from each mouse were surgically removed and pinned at the tendons to a Sylgard coated dish, then incubated for 90–110 min at 35 °C under 5% CO_2_ in a collagenase solution (MEM media containing 1.5 mg/mL Type I collagenase (Sigma Aldrich, St. Louis, MO, USA), 4.5 mg/mL Type II collagenase (Worthington, Lakewood, NJ, USA), and 10% *v*/*v* horse serum (HyClone Laboratories, Inc. Logan, UT, USA). Following collagenase treatment, the fibers were washed four times in a nominally 0 mM Ca^2+^ Tyrode’s solution (mM: 140 NaCl, 5.5 KCl, 10 Hepes free acid, 5.5 d-Glucose, 2 MgCl_2_, pH 7.2) and further dissociated by trituration of superficial fibers with fire-polished glass Pasteur pipettes. After washing and trituration, the dissociated fibers were left an additional 10 min in a nominally Ca^2+^-free Tyrode’s Solution. Up to 200 dissociated fibers were obtained from each animal.

### 4.3. Rb Uptake by Dissociated Intact Fibers Measured using a Multi-Well Format

Rb^+^ uptake by the fibers was measured simultaneously in the various experimental conditions using a multi-well format. Parallel measurements on samples of 2–10 fibers per well and 5–10 wells were made for each experimental condition (24–94 total wells). All samples in a plate were processed using the identical temperature, time after dissection and dissociation, uptake times, and protocol sequence. For kinetic measurements (Figure 6), uptake is initiated simultaneously for all conditions and samples are subsequently harvested in 30 s intervals.

After washing, 5–10 dissociated fibers were transferred to each well using a glass capillary attached to via plastic tubing to a 100 µL pipette. Each well contained 200 μL standard Tyrode’s solution (mM: 140 NaCl, 5.5 KCl, 10 Hepes free acid, 5.5 d-Glucose, 2.5 CaCl_2_, 2 MgCl_2_, pH 7.2). The plate was covered and the fibers were incubated for 30 min at room temperature to allow fibers to attach to the bottom of the plate. Enzymatic dissociation causes some loss of viable fibers. Fibers which become visibly damaged upon return to Ca^2+^-containing Tyrode’s (over-contracted with visibly compromised, rippled membranes) were removed. The remaining fibers were further selected based on having a straight, translucent, smooth surface devoid of kinks, and a tight attachment to the well bottom. Tightly attached fibers were selected by perfusing solution through each well and manually removing any fibers which detached and floated to the surface. This procedure consistently produced viable fibers which took up Rb^+^ in a reproducible manner (RSD of Rb^+^ uptake in the presence of 5 mM Rb^+^ (after normalization) is less than 20% for fibers from the same animal). Approximately 10% of the total number of fibers transferred to the plate remained attached and were used for measurements. These fibers were equilibrated an additional 10 min in standard Tyrode’s solution at 35 °C. Then, ouabain (final concentration: 10 µM or 2 mM) or salbutamol (final concentration: 10 µM) was added to some wells and allowed to bind for 5 min Next, 10 µL RbCl in uptake solution (final Rb^+^ concentrations 0–12 mM) was added to control and test wells to initiate Rb^+^ uptake by the fibers. Fibers were incubated in uptake solution for periods of 30 s–4 min, then washed up to 10× 1 min with ice cold Rb-free wash solution containing (mM): 15 Tris-Cl, 3 BaCl_2_, 2.5 CaCl_2_, 1.2 MgCl_2_, 263 Sucrose, pH 6.8, at 0 °C.

### 4.4. Quantification of Rb^+^ Content by ICP-MS

The amount of Rb^+^ taken up by each sample/well during the incubation period was measured by ICP-MS, as described previously. In brief, each sample was transferred to an acid-washed, metal-free 1 mL clear Eppendorf tube containing 200 µL 1:1 HNO_3_:H_2_O and 0.1 mL of internal standard mix. Samples were digested by heating in an oven for 12 h at 60 °C. After digestion and acid mineralization, sample volumes were brought to 500 µL with doubly deionized water. Samples were introduced to an Agilent 8800 ICP-MS-QQQ (Agilent Technologies, Santa Clara, CA, USA) equipped with a High Efficiency Quartz Concentric Nebulizer, (HEN) (Meinhard, Golden, CO, USA), a Peltier cooled double-pass spray chamber, standard torch, and autosampler. Concentrations of the selected elements (ng/g) were measured by the external calibration method. Data analysis was performed using Mass Hunter workstation version 4.1 (Agilent Technologies, USA). The amount of Rb^+^ taken up by the muscle during the incubation period, after subtraction of the average endogenous Rb^+^ content of untreated fibers from the same animal, was normalized to the P content of the same sample. Rb^+^ uptake rate was expressed as Rb^+^ concentration (ppb) per sample/per P content (ppb). The amount of K^+^ taken up by the Na,K-ATPase was obtained using the molar ratio of Rb/K in the uptake solution. Average transport rates were determined as the amount of Rb^+^ taken up per sample per uptake interval.

### 4.5. ATPase Activity

A plasma membrane fraction enriched in surface sarcolemma and t-tubules was prepared from EDL muscles of WT mice as described previously [8]. In brief, 4–6 EDL muscles (~110 mg) were minced and homogenized using a tissue tearor (Biospec Products) in ice cold homogenization buffer containing (mM): 250 sucrose, 5 EGTA, 30 histidine, protease inhibitor cocktail (Sigma-Aldrich, St. Louis, MO, USA), and 0.1% deoxycholate (pH 6.8). The crude homogenate was centrifuged at 3700× *g* for 15 min (4 °C). The resultant supernatant was centrifuged at 200,000× *g* for 90 min (4 °C). The final pellet containing a plasma membrane-enriched fraction was re-suspended in solubilization buffer (1 mM imidazole, 1 mM EDTA) and used to assay enzyme activity. Protein concentration was measured using BSA Protein Assay Standards (Thermo Fisher Scientific, Waltham, MA, USA).

K^+^-dependent Na,K-ATPase activity was determined from the amount of inorganic phosphate (Pi) released from samples incubated with and without ouabain (2 mM). Samples having 40 μL volume and 1 mg/mL protein were preincubated for 10 min in buffer containing (mM): 80 NaCl, 20 KCl, 5 EGTA, 5 MgCl_2_, 50 Tris-base in a reaction volume of 200 µL. K^+^ or Rb^+^ concentrations were varied by substituting K^+^ or Rb^+^ with choline chloride on an equimolar basis at constant osmolarity. After 15 min equilibration, the reaction was started by addition of solution containing excess ATP (Na salt; final concentration 1 mM). 40 μL of each sample were transferred to 4 wells in a 96-well plate (10 µL each well). All reactions were carried out at 37 °C. The reactions were terminated after 30 min by the addition of 250 µL Biomol Green reagent (BML-AK111-0250; Enzo Life Sciences, Farmingdale, NY, USA). The absorbance at 620 nM was measured (EnVision 2103 Multilabel Plate Reader; Perkin-Elmer, Waltham, MA, USA), and the amount of Pi released was calculated using a standard curve. The amount of Pi released in ouabain-containing buffer was considered nonspecific and deducted from total released Pi. Each sample was measured in quadruplicate and the obtained means were used to compute *V_max_* and the apparent affinities for K^+^ or Rb^+^.

### 4.6. Data Analysis and Statistics

Data were analyzed using Prism (GraphPad Software., 6.07 Version, La Jolla, CA, USA) and reported as the mean value ±SEM. Differences between means were evaluated using the Student’s *t*-test or one-way ANOVA with significance at *p* < 0.05. Welch’s correction was used when SDs were significantly different between compared groups. *K_1/2_* and *V_max_* values were obtained by fitting the measurements of pump activity (Rb^+^ uptake or ATPase hydrolysis) at different K^+^ or Rb^+^ concentrations to the Michaelis–Menten equation:

(1) $$V=V_{max}\;\frac{\left[K^{+}\right]o}{\left[K^{+}\right]o+K_{1/2}}$$

where *V_max_* is the maximum pump activity indicated by Rb^+^ uptake, [K^+^]_o_ is the extracellular K^+^ concentration, and *K_1/2_* is the K^+^ concentration for half-activation of *V_max_*.

One-phase exponential decay regression model was used to analyze the effect of number of washes on Rb^+^ content (Figure 2).

## Figures and Tables

**Table 1:** Components of Rb uptake in myofibers.

	Mean Rb	SEM	Mean Rb/P	SEM
Endogenous Rb	1.589	0.37	0.007	0.002
Passive (+ ouabain)	12.66	4.16	0.028	0.008
Total Rb^+^ uptake	35.72	6.56	0.155	0.026
Stimulate (+ Salbutamol)	65.83	9.50	0.268	0.035

**Table 2:** Maximum turnover rate and affinity constants of Na,K-ATPase for K^+^ and Rb^+^.

	*V_max_*	*K*_1/2_, mM	*n*
K	96.9 ± 47.1	4.79 ± 5.71	31
Rb	64.5 ± 26.6	4.14 ± 4.41	32
*p* value	0.002	0.621	

**Table 3:** Summary of *K*_1/2*,K*_ and *V_max_* obtained from measurements of specific α_1_ or α_2_ activity.

Mouse Model	Treatment	Active NKA Isoform	*K*_1/2_ *,_K_*	*V_max_*	No.
WT	No treatment	α_1_ + α_2_	3.01 mM ± 1.66 mM	0.222 ± 0.035	46
WT	10 µM ouabain	α_1_	1.30 mM ± 1.40 mM	0.088 ± 0.021	39
‘SWAP’	10 µM ouabain	α_2_	3.95 mM ± 2.85 mM	0.181 ± 0.039	46
skα_2_*^−/−^*	No treatment	α_1_	1.02 mM ± 0.95 mM	0.146 ± 0.025	46

**Table 4:** K^+^ affinity of Na,K-ATPase α_1_ and α_2_ isozymes in skeletal muscles determined using four experimental models.

Source	Measurement	Experimental Model	V_m_ (mV)	Na^+^ & K^+^ Conc. Gradients	α_1-_*K*_1/2_*_,K_*_,_ (mM)	α_2-_*K*_1/2*,K*_ (mM)	α_1_ + α_2-_*K*_1/2*,K*_ (mM)
[8]	pump current	voltage-clamped single FDB fibers of WT mice	−90	Yes		4.3 ± 0.3	
[8]	ATPase turnover	sarcolemmal-enriched membranes from hindlimb muscles of WT mice	0	No	1.7 ± 0.6	3.6 ± 0.5	
Figure 4	ATPase turnover	sarcolemmal-enriched membranes from WT mouse EDL	0	No			4.8 ± 5.7
Figure 5	Rb uptake	samples of 2–5 dissociated EDL fibers of WT mice + 10 µM ouabain (α_1_ active) or ‘SWAP’ mice + 10 µM ouabain (α_2_ active)	resting	Yes	1.3 ± 1.4	4.0 ± 2.9	3.0 ± 1.7
Figure 7	Rb uptake	samples of 2-5 dissociated EDL fibers from *skα_2_^−/−^* mice (α_1_ only)	resting	Yes	1.0 ± 0.9		

## Abbreviations

EDL	Extensor digitorum longus
FDB	Flexor digitorum brevis
ICP-MS	Inductively coupled plasma mass spectrometry
ppb	Part per billion
RSD	Relative standard deviation
SEM	Standard error of mean
SD	Standard deviation
SK	Skeletal muscle
